# Gene prioritization and clustering by multi-view text mining

**DOI:** 10.1186/1471-2105-11-28

**Published:** 2010-01-14

**Authors:** Shi Yu, Leon-Charles Tranchevent, Bart De Moor, Yves Moreau

**Affiliations:** 1 Bioinformatics Group, Department of Electrical Engineering, Katholieke Universiteit Leuven, Kasteelpark Arenberg 10, Heverlee B3001, Belgium

## Abstract

**Background:**

Text mining has become a useful tool for biologists trying to understand the genetics of diseases. In particular, it can help identify the most interesting candidate genes for a disease for further experimental analysis. Many text mining approaches have been introduced, but the effect of disease-gene identification varies in different text mining models. Thus, the idea of incorporating more text mining models may be beneficial to obtain more refined and accurate knowledge. However, how to effectively combine these models still remains a challenging question in machine learning. In particular, it is a non-trivial issue to guarantee that the integrated model performs better than the best individual model.

**Results:**

We present a multi-view approach to retrieve biomedical knowledge using different controlled vocabularies. These controlled vocabularies are selected on the basis of nine well-known bio-ontologies and are applied to index the vast amounts of gene-based free-text information available in the MEDLINE repository. The text mining result specified by a vocabulary is considered as a view and the obtained multiple views are integrated by multi-source learning algorithms. We investigate the effect of integration in two fundamental computational disease gene identification tasks: gene prioritization and gene clustering. The performance of the proposed approach is systematically evaluated and compared on real benchmark data sets. In both tasks, the multi-view approach demonstrates significantly better performance than other comparing methods.

**Conclusions:**

In practical research, the relevance of specific vocabulary pertaining to the task is usually unknown. In such case, multi-view text mining is a superior and promising strategy for text-based disease gene identification.

## Background

Text mining helps biologists to collect disease-gene associations automatically from large volumes of biological literature. During the past decade, there was a surge of interests in automatic exploration of the biomedical literature, ranging from modest approaches such as annotating and extracting keywords from biomedical text to more ambitious attempts like Natural Language Processing (NLP), text-based network construction and inference, and so on. These computational efforts effectively help biologists to identify the most likely disease candidates for further experimental validation. Currently, the most important resource for biomedical text mining applications is the MEDLINE database developed by the National Center for Biotechnology Information (NCBI) at the National Library of Medicine (NLM). MEDLINE covers all aspects of biology, chemistry, and medicine, there is almost no limit to the types of information that may be recovered through careful and exhaustive mining [1]. To extract relevant *information *out of the immense amount of *data*, furthermore, to retrieve useful high-level *knowledge *from the *information*, *text mining *and *machine learning *have become indispensable tools in practical research.

Nevertheless, the selection of controlled vocabularies and the representation schemes of terms occupy a central role in *text mining *and the efficiency of knowledge discovery varies greatly between different text mining models [2]. To address these challenges, we propose a *multi-view text mining *approach to retrieve information from different biomedical domain levels and combine it to identify disease relevant genes in prioritization and clustering. Our notion of *view *is a text mining model specified by a controlled vocabulary (CV), so the concept of *multi-view *text mining is featured with combining multiple controlled vocabularies to retrieve gene-centric perspectives from free text publications. It also implies that all the information is retrieved from the identical MEDLINE corpus but varies by the adopted vocabulary, so the term *view *denotes a domain-based perspective of the corpus. The idea of *multi-view *perspectives is well-known in modern mechanical design and drawing, where a mechanical component is illustrated as three or more views using the the applied geometry method developed by Gaspard Monge in the 1780s [3]. In the context of genomic data fusion, the idea of incorporating more views in analysis may be beneficial, by reducing the noise, as well as improving statistical significance and leveraging the interactions and correlations between the text mining models to obtain more refined and higher-level information [4].

To retrieve *multi-view *information, we select multiple vocabularies on the basis of nine bio-ontologies (GO, MeSH, eVOC, OMIM, LDDB, KO, MPO, SNOMED-CT, and UniprotKB). The text mining process (i.e., vocabulary selection, literature indexing, creation of gene-by-term profiles, etc.) is similar to our earlier work investigating performance of a single text mining model in gene prioritization [2]. The novel aspect of the present approach is the combination of multiple text models in a joint framework of data fusion and dimensionality reduction. Moreover, we extend the task from disease gene prioritization to clustering and propose several new algorithms for data fusion based clustering analysis. It also emphasizes our text mining approach as a general method, whose resulting high dimensional gene-by-term profiles are adoptable in a wide-range of computational tasks such as prioritization, clustering, classification analysis, and so on. Furthermore, the gene-by-term profiles obtained in our approach can also be combined with biological data thus the discovery of disease associated genes is balanced between reliability and novelty.

Beside the motivation of the *multi-view *approach, how to combine models for better performance with the multiplicity of *machine learning *methodologies still remains a challenge. Some related work [5-7] has proposed to incorporate text mining models using basic operators such as arithmetic average, max, min, union and so on. Unfortunately, our preliminary experiment [2] shows that these basic operators may work in some circumstances, but cannot guarantee the superiority of the combined model. To explore an effective methodology to integrate the *multi-view *data, we systematically compare the performance of several integration methods on real disease benchmark data. These integration methods not only consist of the basic operators mentioned before but also include some advanced machine learning techniques for data fusion, such as consensus functions, multiple kernel learning algorithms, and so on. Moreover, to tackle the high dimensionality of text mining results, we introduce dimensionality reduction techniques in the previously developed data fusion framework. Two different dimensionality reduction approaches are applied: latent semantic indexing (LSI) and vocabulary pruning with ontological structure. The former one is a well established method in information retrieval. The latter one is a specific method to select the indexed terms according to the hierarchical structure of bio-ontologies. Beside the *multi-view *strategy, we also try some other alternative approaches: one approach is to merge different numbers of CVs as a union vocabulary for text mining; another approach is to further resolve the heterogeneities of terms in the union vocabulary by mapping them into unique concepts. The performance of these alternative approaches is also compared and the advantage of the proposed *multi-view *is clearly demonstrated.

We investigate two fundamental tasks in disease associated gene research: prioritization and clustering. Both tasks have attracted lots of efforts in the literature, whereas their definitions and interpretations may vary by approach. Their computational definitions are clarified in the METHOD section of the present paper. Genome-wide experimental methods to identify disease causing genes, such as linkage analysis and association studies, often produce large sets of candidate genes [8]. On one hand, computational gene prioritization methods *rank *the large amount of candidate disease genes according to their likeliness of being involved in a certain disease. On the other hand, clustering analysis explores the disease-gene associations by partitioning the genes based on the experimental findings described in the scientific literature. These two tasks basically share a similar assumption: In prioritization, the similarity among genes associated to the same disease is assumed to be higher than the similarity with random genes. In the case of multiple diseases, the problem can also be formulated as a clustering problem. The assumption is that the similarity of genes relevant to the same disease (within-disease-cluster similarity) is higher than the similarity of genes relevant to different diseases (between-disease-cluster similarity). Thus, we expect these genes to demonstrate some "natural partitions" according to the type of diseases. Therefore, we are able to evaluate the performance of prioritization task and the clustering task using the same disease benchmark data.

As mentioned, given multiple data sources, to obtain an effective combined model in the prioritization and the clustering tasks is still a non-trivial issue. The data for prioritization only contains positive samples and the clustering data are all unlabeled samples, so it is often hard to validate each individual model and select the best models for integration. Furthermore, text mining is often used by biologists as an explorative method to gain first-hand knowledge about the associations of genes with disease, usually there is limited amount of prior knowledge for model evaluation. To tackle this difficulty, the integration methods proposed in this paper do not rely on evaluation of individual models. Based on related work, we review and categorize several algorithms as two general strategies, ensemble learning and kernel fusion, to combine multiple models. The proposed methods are shown effective, also robust, when combining relevant and irrelevant models. The performance of combination is shown significantly better than the best individual model, moreover, is also comparable to the ideal performance obtained by combining best models only. To explain why the improvements take place, we present case studies to investigate the false positive genes in prioritization and the mis-classified genes in clustering.

## Methods

### Selection of controlled vocabularies from multiple bio-ontologies

We select vocabularies from nine bio-ontologies for text mining, among which five of them (GO, MeSH, eVOC, OMIM and LDDB) have proven their merit in our earlier work of text based gene prioritization [2] and text based cytogenetic bands mapping [9]. Besides these five, we select four additional ontologies (KO, MPO, SNOMED CT, and UniprotKB) because they are also frequently adopted in the identification of genetic diseases and signaling pathways, for instance, in the works of Gaulton *et al*. [5], Bodenreider [10], Mao *et al*. [11], Smith *et al*. [12], and Melton *et al*. [13]. The nine bio-ontlogies are briefly introduced as follows.

#### The Gene Ontology

GO [14] provides consistent descriptions of gene and gene-product attributes in the form of three structured controlled vocabularies that each provide a specific angle of view (biological processes, cellular components and molecular functions). GO is built and maintained with the explicit goal of applications in text mining and semantic matching in mind [9]. Hence, it is an ideal source as domain-specific views in our approach. We extract all the terms in GO (due to the version released in December, 2008) as the CV of GO.

#### Medical Subject Headings

MeSH is a controlled vocabulary produced by NLM for indexing, cataloging, and searching biomedical and health-related information and documents. The descriptors or subject headings of MeSH are arranged in a hierarchy. MeSH covers a broad range of topics and its current version consists of 16 top level categories. Though most of the articles in MEDLINE are already manually annotated with MeSH terms, our text mining process does not rely on these annotations but indexes the MEDLINE repository automatically with the MeSH descriptors (version 2008).

#### Online Mendelian Inheritance in Man's Morbid Map

OMIM [15] is a database that catalogues all the known diseases with genetic components. It contains available links between diseases and relevant genes in the human genome and provides references for further research and tools for genomic analysis of a catalogued gene. OMIM is composed of two mappings: the OMIM Gene Map, which presents the cytogenetic locations of genes that are described in OMIM; the OMIM Morbid Map, which is an alphabetical list of diseases described in OMIM and their corresponding cytogenetic locations. Our approach retrieves the disease descriptions from the OMIM Morbid Map (version due to December, 2008) as the CV.

#### London Dysmorphology Database

LDDB is a database containing information over 3000 dysmorphic and neurogenetic syndromes, which is initially developed to help experienced dysmorphologists to arrive at the correct diagnosis in difficult cases with multiple congenital anomalies [16]. Information in the database is constantly updated and over 1000 journals are regularly reviewed to ascertain appropriate reports. The London Neurology Database (LNDB) is a database of genetic neurological disorders based on the same data structure and software as the LDDB [17]. We extract the dysmorphology taxonomies from LNDB (version 1.0.11) and select the vocabulary terms.

#### eVOC

eVOC [18] is a set of vocabularies that unifies gene expression data by facilitating a link between the genome sequence and expression phenotype information. It was originally categorized as four orthogonal controlled vocabularies (anatomical system, cell type, pathology, and developmental stage) and now extended into 14 orthogonal subsets subsuming the domain of human gene expression data. Our approach selects the vocabulary from the eVOC version 2.9.

#### KEGG Orthology

KO is a part of the KEGG suite [19] of resources. KEGG is known as a large pathway database and KO is developed to integrate pathway and genomic information in KEGG. KO is structured as a directed acyclic graph (DAG) hierarchy of four flat levels [11]. The top level consists of the following five categories: metabolism, genetic information processing, environmental information processing, cellular processes and human diseases. The second level divides the five functional categories into finer sub-categories. The third level corresponds directly to the KEGG pathways, and the fourth level consists of the leaf nodes, which are the functional terms. In literature, KO has been used as an alternative controlled vocabulary of GO for automated annotation and pathway identification [11]. The KO based controlled vocabulary in our approach is selected on the version due to December 2008.

#### Mammalian Phenotype Ontology

MPO [20] contains annotations of mammalian phenotypes in the context of mutations, quantitative trait loci and strains which was initially used in Mouse Genome Database and Rat Genome Database to represent phenotypic data. Because mouse is the premier model organism for the study of human biology and disease, in the CAESAR [5] system, MPO has also been used as a controlled vocabulary for text mining based gene prioritization of human diseases. The MPO based controlled vocabulary in our approach is selected on the version due to December 2008.

#### Systematized Nomenclature of Medicine-Clinical Terms

SNOMED is a huge and comprehensive clinical terminology, originally created by the College of American Pathologists and, now owned, maintained, and distributed by the International Health Terminology Standards Development Organization (IHTSDO). SNOMED is a very "fine-grained" collection of descriptions about care and treatment of patients, covering areas like diseases, operations, treatments, drugs, and healthcare administration. SNOMED has been investigated as an ontological resource for biomedical text mining [10] and also has been used in patient-based similarity metric construction [13]. We select the CV on the SNOMED (version due to December, 2008) obtained from the Unified Medical Language System (UMLS) of NLM.

#### Universal Protein Knowledgebase

UniProtKB [21] is a repository for the collection of functional information on proteins with annotations developed by European Bioinformatics Institute (EBI). Annotations in UniProtKB are manually created and combined with non-redundant protein sequence database, which brings together experimental results, computed features and scientific conclusions. Mottaz *et al*. [22] design a mapping procedure to link the UniProt human protein entries and corresponding OMIM entries to the MeSH disease terminology. The vocabulary applied in our approach is selected on UniProt release 14.5 (due to December, 2008).

The terms extracted from these bio-ontologies are stored as bag-of-words and preprocessed for text mining. The preprocessing includes transformation to lower case, segmentation of long phrases, and stemming. After preprocessing, these vocabularies are fed into a Java program based on Apache Java Lucene API to index the titles and abstracts of MEDLINE publications relevant to human genes.

### Vocabularies selected from subsets of ontologies

As mentioned, in some "fine-grained" bio-ontologies the concepts and terminologies are labeled in multiple hierarchies, denoted as sub-ontologies, to represent domain concepts at various levels of specificity. For instance, GO consists of three sub-ontologies: biological process, cellular component and molecular function. MeSH descriptors are arranged in 16 hierarchical trees at the top level. In SNOMED, the medical terminologies are composed of 19 higher level hierarchies. eVOC ontology contains 14 orthogonal vocabulary subsets, whose terms contained are strictly non-overlapping. To investigate whether more specific vocabularies can improve the effectiveness of the text mining model, we select terms from the sub-ontologies of GO, MeSH, SNOMED, and eVOC and compose the corresponding subset CVs. Considering the main objective as disease-associated gene identification, only the most relevant sub-ontologies (6 from eVOC, 7 from MeSH and 14 from SNOMED) are selected. Other sub-ontologies either have very few terms or have no relation with the topic of disease gene identification so they are not selected. To distinguish the gene-by-term profiles obtained from subset CVs from those obtained from complete CVs, we denote the former ones as subset CV profiles and the latter ones as complete CV profiles.

### Merging and mapping of controlled vocabularies

The strategy of incorporating multiple CVs may be alternatively achieved by merging terms of several vocabularies together. To investigate this, we merge the terms of all 9 complete CVs as a union of vocabulary and denote the corresponding gene-by-term text mining result as "merge-9 profile". Furthermore, we notice the lexical variants across multiple ontologies: a concept may be represented as different terms due to the diversities of professional expressions. For example, the MeSH term "denticles" is expressed as "dental pulp stones" in OMIM and as "pulp stone" in SNOMED. To resolve these variants, we refer to the UMLS Metathesaurus to map terminological variants as unified concepts. We download the concept names and sources file (MRCONSO.RRF) from UMLS Metathesaurus, which provides the mapping of atoms (each occurrence of unique string or concept name within each source vocabulary) to unified concepts. In text mining, we build a synonym vocabulary to map and aggregate the occurrences of various synonym terms as the occurrences of the unified concepts. The obtained results are gene-by-concept profile whose features are the unique and permanent concept identifiers defined by UMLS Metathesaurus. Among the nine vocabularies adopted in our approach, only four of them (GO, MeSH, OMIM and SNOMED) are included in UMLS and resolved in the MRCONSO.RRF file. Therefore, to fairly compare the effect of concept mapping, we also create "merge-4" gene-by-term profile using the union of the four vocabularies in indexing. Then, we map the lexical variants as concepts and the result is denoted as "concept-4" profile. Moreover, to create a naive baseline, we also index the MEDLINE corpus without using any controlled vocabulary. All the terms appeared in the corpus are segmented as single words and the results are expressed by these vast amount of words, denoted as "no-voc profile". We didn't consider the phrases of multiple words because there would be an immense number of combinations.

### Text mining

We create the gene-by-term profiles according to the mapping of genes and publications in Entrez GeneRIF. A subset of MEDLINE literature repository (as of 10 December, 2008) that consists of 284,569 human gene-related publications is selected for text mining. In the first step all these 284,569 MEDLINE documents are indexed and the doc-by-term (or doc-by-concept) vectors are constructed. In the second step, we averagely combine the document-by-term (document-by-concept) vectors as gene-by-term (gene-by-concept) vectors according to the GeneRIF mapping. The detail of the text mining process is presented in our earlier work [2,23]. Table 1 lists all the CVs applied in our approach. Table 2 illustrates the overlapping terms among the nine complete CVs.

**Table 1:** Overview of the controlled vocabularies applied in the multi-view approach.

**No**.	CV	Number of terms in CV	Number of indexed terms
1	**eVOC**	1659	1286
2	eVOC anatomical system	518	401
3	eVOC cell type	191	82
4	eVOC human development	658	469
5	eVOC mouse development	369	298
6	eVOC pathology	199	166
7	eVOC treatment	62	46
8	**GO**	37069	7403
9	GO biological process	20470	4400
10	GO cellular component	3724	1571
11	GO molecular function	15282	3323
12	**KO**	1514	554
13	**LDDB**	935	890
14	**MeSH**	29709	15569
15	MeSH analytical	3967	2404
16	MeSH anatomy	2467	1884
17	MeSH biological	2781	2079
18	MeSH chemical	11824	6401
19	MeSH disease	6717	4001
20	MeSH organisms	4586	1575
21	MeSH psychiatry	1463	907
22	**MPO**	9232	3446
23	**OMIM**	5021	3402
24	**SNOMED**	311839	27381
25	SNOMED assessment scale	1881	810
26	SNOMED body structure	30156	2865
27	SNOMED cell	1224	346
28	SNOMED cell structure	890	498
29	SNOMED disorder	97956	13059
30	SNOMED finding	51159	3967
31	SNOMED morphologic abnormality	6903	2806
32	SNOMED observable entity	11927	3119
33	SNOMED procedure	69976	9575
34	SNOMED product	23054	1542
35	SNOMED regime therapy	5362	1814
36	SNOMED situation	9303	2833
37	SNOMED specimen	1948	742
38	SNOMED substance	33065	8948
39	**Uniprot**	1618	520
40	Merge-9	372527	50687
41	Merge-4	363321	48326
42	Concept-4	1420118	44714
43	No-voc	-	259815

The versions of bio-ontologies and MEDLINE repository adopted in the indexing process are mentioned in the text. The *Number of indexed terms *of controlled vocabularies reported in this table are counted on indexing results of human related publications only so their numbers are smaller than those in our earlier work [2], which were counted on all species appeared in GeneRIF. The *Number of terms in CV *are counted on the vocabularies independently from the indexing process. The numbers of terms of Merge-9, Merge-4 and Concept-4 are counted on text mining results of all species occurring in GeneRIF.

**Table 2:** The number of overlapping terms in different vocabularies and indexed terms

		eVOC	GO	KO	LDDB	MeSH	MPO	OMIM	SNOMED	Uniprot
		**1659**	**37069**	**1514**	**935**	**29709**	**9232**	**5021**	**311839**	**1618**

eVOC	1286	-	370	16	118	827	566	325	876	46
GO	7403	358	-	404	74	3380	1234	659	4772	325
KO	554	16	344	-	1	383	72	120	489	44
LDDB	890	118	74	1	-	346	275	205	498	16
MeSH	15569	784	2875	344	343	-	2118	1683	12483	373
MPO	3446	554	1177	72	271	2007	-	823	2729	146
OMIM	3402	322	655	119	205	1644	816	-	2275	161
SNOMED	27381	814	3144	380	492	8900	2508	2170	-	593
Uniprot	520	46	301	42	16	361	146	157	371	-

The upper triangle matrix shows the numbers of overlapping terms among vocabularies independent from indexing. The lower triangle matrix shows the numbers of overlapping indexed terms. The second horizontal row (from the top) are the numbers of the terms in vocabularies independent from indexing. The second vertical column (from the left) are the numbers of the indexed terms.

Preliminary result shows that the weighting scheme of terms also influences the performance of gene-by-term data in biological validation [2]. When the same vocabulary and the ranking algorithm are applied in prioritization, the IDF representation generally outperforms the TF-IDF and the binary representations. Therefore, in this article all the term profiles are represented in the IDF weighting scheme.

### Dimensionality reduction of gene-by-term data by Latent Semantic Indexing

We have introduced the subset CVs method to reduce the number of terms expressing the genes. Alternatively, we also apply LSI to reduce the number of term features. On the one hand, the information expressed on vast numbers of terms is mapped to a smaller number of latent factors so the irrelevant information is reduced. On the other hand, we expect that LSI does not compromise the information required for prioritization and clustering. In implementation, we use the Matlab function *svds *to solve the eigenvalue problem of the sparse gene-by-term matrix of the whole human genome (22,743 human genes). To calculate the total variance on this huge matrix is very computational expensive, so we sort the eigenvalues obtained by the sparse eigenvalue decomposition. To determine the number of latent factors, we select the dimension where the corresponding smallest eigenvalue is less than 0.05% of the sum of all eigenvalues.

### Algorithms and evaluation of gene prioritization task

The computational definition of gene prioritization is mentioned in our earlier work [2,24-26]. We briefly introduce it here for completeness. Genes that are known relevant to the same disease are constructed as a disease-specific training set. A prioritization model is first built on this training set, then that model is used to rank a test set of candidate genes according to their similarity to the model. The performance is evaluated by checking the positions of the real relevant genes in the ranking of a test set. A perfect prioritization should rank the gene with the strongest causal link to the biomedical concept, represented by the training set, at the highest position (at the top). The interval between the real position of that gene and the top is similar to the error. For a prioritization model, minimizing this error is equal to improving the ranking position of the most relevant gene and in turn it reduces the number of irrelevant genes to be investigated in biological experimental validation. So a model with smaller error is more efficient and accurate to find disease relevant genes and that error is also used as a performance indicator for model comparison [2]. The ranking of candidate genes is usually based on scores. Assuming a larger score represents a higher similarity towards the prioritization model, in benchmark study, one can label the real relevant genes as class "+1" and other irrelevant genes as class "-1" and plot the Receiver operating characteristic (ROC) curves to compare different models by the values of area under curve (AUC). The error of prioritization is thus equivalent to 1 minus the AUC value.

The methods to combine models for prioritization are roughly classified as two approaches: ensemble of rankings and fusion of sources (kernels).

#### Ensemble ranking

In ensemble ranking, the prioritization is first carried on each individual model and then multiple ranking results are combined. Since our main objective is to integrate the models, we use the same algorithm, standard correlation (Pearson's correlation) [27], as the base method to obtain ranking results on individual models. Using other algorithms, the results after model integration may not be significantly different and the computational complexity is more likely to be higher than the standard correlation algorithm.

The ranking results are integrated either as ranking orders, ranking ratios (the order of ranking divided by the total number of ranking candidates) or ranking scores. To compare them, we implement three integration algorithms. Two of them are basic operators to calculate the average or maximal value of multiple ranking scores. The third one is based on order statistics to combine ranking orders, as implemented in the Endeavour system [24,26]. The order statistics is formulated as follows: For each gene, a Q statistics is calculated from all rank ratios using the joint cumulative distribution of an N-dimensional order statistic as previously done by Stuart *et al*. [28], given by(1)

where *r*_*i *_is the rank ratio for data source *i*, *N *is the number of data models. To reduce the complexity, Aerts *et al*. [24] implement a much faster alternative formula to approximate the integration, given by:(2)

where(3)

where *r*_*i *_is the rank ratio for data source *i*, *r*_*N*-*k*+1 _is the rank ratio for data source *N *- *k *+ 1 and *V*_0 _= 1.

The Q statistics for randomly and uniformly drawn rank ratios is found approximately distributed according to a beta distribution when *N *≤ 5, and a gamma distribution for *N *> 5. According to the cumulative distribution, we obtain P-value for the Q value computed as (2) [24] for each gene. Thus the original *N *rankings are combined into a ranking of P-values computed for each gene.

#### Kernel fusion for prioritization

The kernel fusion method for gene prioritization is proposed by De Bie *et al*. [25] as a one-class SVM (1-SVM) problem, where the kernels derived from multiple sources are combined in a weighted convex form, given by(4)

where *K*_*j *_is the *j*-th centered kernel matrix of training genes plus candidate genes, *μ*_*j *_is the weighting coefficient on *K*_*j*_, *r*_*j *_is a regularization parameter controlling the complexity of kernels and is set as the trace value of *K*_*j *_and *N *is the number of data sources. The objective is to maximize the margin between the origin point and the separating hyperplane defined by the integrated model. The problem is solved as a quadratic constraint linear programming (QCLP) problem, given by(5)(6)

where *ν *is the regularization parameter penalizing part of the training data as outliers, M is the number of training genes, *α*_*i *_are the dual variables, *G*_*j *_is the kernel matrix of training genes taken from *K*_*j*_, *t *is the dummy variable for optimization. In this QCLP formulation, the weighting coefficients *μ*_*j *_are corresponding to the dual variables bounded by the constraints in (6) and their sum is equal to 1.

We apply this 1-SVM method to combine kernels derived from the multi-view data for gene prioritization. Because the dimensionality of gene-by-term profile is high, we only use linear function to construct the kernel matrices. One of the main features of the 1-SVM is the sparsity of its solutions, whose dominant coefficient may be assigned on one or two kernels. This property is useful to distinguish a small amount of relevant sources from a large number of irrelevant sources in data fusion. However, in biomedical applications, the data sources are usually preprocessed and have high relevances w.r.t. the problem. Sparse solution may be too selective, in this case, to thoroughly combine the redundant and complementary information in these data sources. To balance the effect of sparse coefficients (most of *μ*_*j *_are equal to 0) and non-sparse coefficients in model generalization, we try 3 different values of the regularization parameter *μ*_*min *_to restrict the optimization process as a lower bound of coefficient assigned on each kernel. When *μ*_*min *_= 0, there is no lower bound and the optimization procedure will probably result in the sparse coefficients. When *μ*_*min *_= 0.5/*N*, each kernel is insured to have a minimum contribution in data fusion. When *μ*_*min *_= 1/*N*, the kernels are averagely combined.

After solving a and *μ*_*j *_in (5), the function applied to prioritize candidate genes is given by(7)

where Ω is optimal combination of kernel defined in (4), *K*_*j*_(*x*, *x*_*i*_) are values of *K*_*j *_where *x *denotes a candidate gene and *x*_*i *_denotes the *i*-th training gene. The score obtained from (7) ranges from -1 to +1 and larger score represents stronger similarity towards the prioritization model. In our implementation, the QCLP problem in (5) is solved by SeDuMi 1.2 [29].

#### Evaluation of prioritization

The prioritization result is evaluated by leave-one-out (LOO) method [2]. In each experiment, given a disease gene set which contains *K *genes, one gene, termed the "defector" gene, is deleted from the set of training genes and added to 99 randomly selected test genes (test set). We use the remaining *K *- 1 genes (training set) to build our prioritization model. Then, we prioritize the test set which contains 100 genes by the trained model and determine the ranking of that defector gene in test data. The prioritization performance is thus measured by the error (1 minus the AUC value).

#### Algorithms and evaluations of gene clustering task

**Figure 1:**
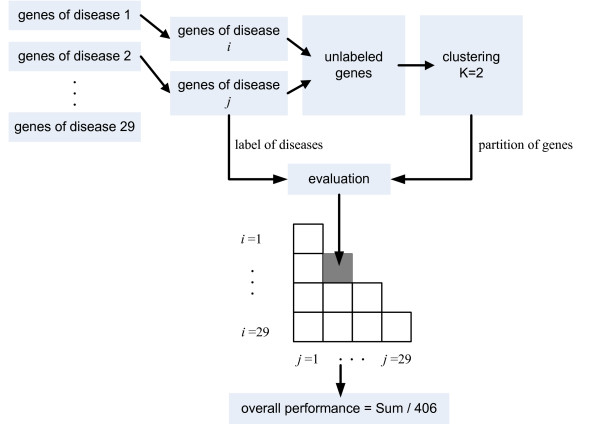
**Conceptual scheme of clustering disease relevant genes**. Using these gene-by-term profiles, we evaluate the performance of clustering a benchmark data set consisting 620 disease relevant genes categorized in 29 genetic diseases. The numbers of genes categorized in the diseases are very imbalanced, moreover, some genes are simultaneously related to several diseases. To obtain meaningful clusters and evaluations, we enumerate all the pairwise combinations of the 29 diseases (406 combinations). For each time, the relevant genes of each paired diseases combination are selected and clustered into two groups, then the performance is evaluated using the disease labels. The genes which are relevant to both diseases in the paired combination are removed before clustering (totally less then 5% genes have been removed). Finally, the average performance of all the 406 paired combinations is used as the overall clustering performance.

As mentioned, the gene-disease associations can be alternatively investigated by segregating the genes into different groups. The within-grouping and between-grouping of genes give useful evidence about their functions and processes in the biological side. Clustering analysis is a fundamental technique to gain this insight and it has been the workhorse for a wide range of applications, such as microarray expression data analysis, protein interaction network analysis, and many others. The clustering of disease relevant genes belongs to another paradigm than prioritization. Firstly, the gene profiles are collected, appropriately preprocessed and poured into a representation. Secondly, a distance measure should be chosen to quantify the relationship between genes, which is usually selected as Euclidean distance or Mahalanobis distance. Thirdly, clustering algorithms are applied on the distance matrix of genes to segregate them into different partitions. Fourthly, the assessment of cluster quality is done based on the data that generated the partitions (i.e., "statistically") or using external information (i.e., "biologically") [30]. The first type of assessment is also known as the internal validation and the second type is called the external validation. The statistical assessment of the data fusion based clustering is still an ongoing issue because most of the internal validation indices (i.e., Silhouette index, Jaccard index, Modularity, etc.) are data dependent, which makes it difficult to evaluate the clustering results among inconsistent indices. Our approach mainly focuses on the biological assessment of clustering and the conceptual scheme of our clustering approach is illustrated in Figure 1.

Analogous to the prioritization task, the methods of data fusion based clustering can also be categorized in two approaches: ensemble clustering and kernel fusion.

##### Ensemble clustering

In ensemble clustering, we apply K-means clustering using Euclidean distance as the "base clustering algorithm" on a single data source to obtain the partition; then we combine the multiple partitions as a consolidate partition via consensus functions. We also tried other candidate algorithms (i.e., hierarchical clustering, self-organizing maps, etc.) and other distance measures (i.e., Mahalanobis distance, Minkowski distance, etc.), although we observe some discrepancies of performance on individual gene-by-term data, the difference after multi-view integration is not significant. In literature, various consensus functions have been proposed for ensemble clustering. We select 6 popular ones and compare them in our approach.

##### CSPA, HGPA, and MCLA

Strehl and Ghosh [31] formulate the optimal consensus as the partition that shares the most information with the partitions to combine, as measured by the Average Normalized Mutual Information. They use three heuristic consensus algorithms based on graph partitioning, called Cluster based Similarity Partition Algorithm (CSPA), Hyper Graph Partitioning Algorithm (HGPA) and Meta Clustering Algorithm (MCLA) to obtain the combined partition.

##### QMI

Topchy *et al*. [32] formulate the combination of partitions as a categorical clustering problem. In their approach, a category utility function is adopted to evaluate the quality of a "median partition" as a summary of the ensemble. They prove that maximizing this category utility function implies the same clustering ensemble criterion as maximizing the generalized mutual information based on quadratic entropy (QMI). Furthermore, the maximization of the category utility function is equivalent to the square error based clustering criterion when the number of clusters is fixed. The final consensus partition is obtained by applying the K-Means algorithm on the feature space transformed by the category utility function.

##### EACAL

Fred and Jain [33] introduce the concept of Evidence Accumulation Clustering (EAC) that maps the individual data partitions as a clustering ensemble by constructing a co-association matrix. The entries of the co-association matrix are interpreted as votes on the pairwise co-occurrences of objects, which is computed as the number of occurrences each pair of objects appears in the same cluster of an individual partition. Then the final consensus partition is obtained by applying single linkage (SL) and average linkage (AL) algorithms on the co-association matrix. According to their experiments, average linkage performs better than single linkage so in this paper we apply Evidence Accumulation Clustering with average linkage (EACAL) as the representative algorithm for comparison.

##### AdacVote

Ayad and Kamel [34] propose an Adaptive cumulative Voting (AdacVote) method to compute an empirical probability distribution summarizing the ensemble. The goal of this ensemble is to minimize the average squared distance between the mapped partitions and the combined partition. The cumulative voting method seeks an adaptive reference partition and incrementally updates it by averaging other partitions to relax the dependence of the combined partition on the selected reference. In the AdacVote they proposed, the partitions are combined in the decreasing order of their entropies.

##### Kernel fusion for clustering

An alternative approach to combine multi-view data for clustering is achieved by fusing the similarity matrices [35], as known as the *kernel fusion *approach. Kernel fusion integrates data before clustering (early integration), whereas ensemble clustering aggregates partitions after clustering (late integration). We implement 5 kernel fusion algorithms and cross-compare their performance. In the present paper, the kernel matrices are all constructed by linear functions because the text mining data is in very high dimension.

##### Hierarchical clustering

We average the kernels of multi-view data and transform it into a distance matrix in Hilbert space, given by [36]:(8)

When the kernel mapping *ϕ *(·) is based on a linear function and *x *and *z *are data vectors normalized by the norm, *d*_*ϕ *_(*x*, *z*) boils down to the Euclidean distance between *x *and *z*. When *ϕ *(·) is based on nonlinear mapping (i.e., RBF functions, Polynomial functions, etc.), the distance *d*_*ϕ *_(*x*, *z*) does not have direct interpretation in the original space of *x *and *z*. Given the distance matrix, we can apply linkage methods (i.e., single linkage, complete linkage, average linkage, and ward linkage) and obtain the hierarchical clustering result in Hilbert space.

##### OKKC

Optimized data fusion for Kernel K-means clustering (OKKC) is proposed in our earlier work [37] as a weighted kernel fusion algorithm for clustering. In OKKC, the convex kernel combination is optimized for clustering by maximizing the between cluster Mahalanobis distance in Hilbert space. OKKC consists of two iteration steps: kernel K-means clustering and kernel fusion. The clustering step obtains class labels of the data; the kernel fusion step optimizes the coefficient of kernels with the given labels. These two steps iterate until convergence. In our implementation, the clustering step is based on Girolami's kernel K-means clustering algorithm [38]. The kernel fusion step is solved by a quadratic constraint quadratic programming (QCQP) problem, given by(9)

where *β*_*j *_is the dual variable of the objective of clustering (not shown here) discriminating the *j*-th class with other classes, *λ *is the regularization parameter smoothing the covariance matrix defined in the Mahalanobis distance, *l*_*j *_is the vector of labels discriminating the *j*-th class with other classes, *k *is the number of classes, *t *is the dummy variable for optimization, *K*_*i *_is the centered kernel matrix of the *i*-th data source, *r*_*i *_is the trace of *K*_*i*_. Analogue to the 1-SVM algorithm in prioritization, we adjust the lowerbound of the coefficients assigned on different kernels via a regularization parameter *μ*_*min*_. Two values are set for *μ*_*min*_: 0 and 1/N. When *μ*_*min *_= 1/*N*, the OKKC algorithm is equivalent to the Girolami's kernel K-means clustering applied on averagely combined kernels. The QCQP problem in (9) is solved by MOSEK toolbox [39]. The computational burden of QCQP problem can be much simplified as Semi-infinite Linear Programming (SILP) formulation, which is inspired by the work of Sonnenburg *et al*. [40] and Ye *et al*. [41]. The technical discussion of this simplification is irrelevant to the topic of this paper, so we use the conventional QCQP formulation here.

##### Evaluation of clustering

As explained before, we assess the clustering performance biologically by labeled disease benchmark data. Two external validations, Rand Index (RI) [42] and Normalized Mutual Information (NMI) [31], are applied and their definitions are given as follows. Given a set of *N *genes *X *= {*x*_1_, ..., *x*_*N *_} and two partitions to compare,  = {*c*_1_, ..., *c*_*N*_} and   = {*p*_1_, ..., *p*_*N*_}. In our problem,  and  are respectively the cluster indicators and the disease labels of the set of genes *X*. We refer that (1) *a*, the number of pairs of genes in *X *that are in the same set in  and in the same set in ; (2) *b*, the number of pairs of genes in *X *that are in different sets in  and in different sets in ; (3) *c*, the number of pairs of genes in *X *that are in the same set in  and in different sets in ; (4) *d*, the number of pairs of genes in *X *that are in different sets in  and in the same set in .

RI is defined as(10)

For binary class problem, the RI value ranges from 0 to 1 and the value of random partitions is 0.5.

NMI is defined as(11)

where *M*(, ) is the mutual information between the indicators, *E*() and *E*() are the entropies of the indicators and the labels. For a balanced clustering problem, if the indicators and the labels are independent, the NMI value approaches 0.

### Benchmark data set of disease genes

We validate the clustering results with the human disease benchmark data set of Endeavour [24], which consists of 620 relevant genes from 29 diseases. Genes from the same disease are constructed as a disease-specific training set used to evaluate the prioritization and clustering performance. For prioritization, we perform 620 rankings (with each gene left out once) on 99 candidate human genes randomly selected from the human genomic. The prioritization is repeated 20 times (with randomly permuted 99 random genes each time for each left out gene) and the average Error value is reported as the final result. For clustering, we enumerate all the paired combinations of the 29 diseases and result in 406 binary clustering tasks. In each paired combination, the overlapping genes (always less than 5% amount of the genes for clustering) are removed for each clustering task. All the 406 tasks are repeated 20 times and the mean value of RI and NMI of all tasks in all repetitions is reported as the final result. The schema of the clustering evaluation is depicted in Figure 1.

## Results

### Multi-view performs better than single view

In prioritization and clustering, integration of *multi-view *data obtained significantly better performance than the best single view data. As presented in Table 3, the best performance in prioritization was obtained by combining 9 complete CV profiles as kernels in the LSI reduced dimensionality (1-SVM+LSI, Error of AUC = 0.0335). This error was only half of the best single CV profile (LDDB, 0.0792) without integration. In clustering, as shown in Table 4, three algorithms obtained significant performance. Ward linkage applied on average combination of kernels showed the best performance (RI = 0.8236, NMI = 0.6015). EACAL (RI = 0.7741, NMI = 0.5542) and OKKC without regularization (*μ*_*min *_= 0, RI = 0.7641, NMI = 0.5395) also performed better than the best single view data (LDDB, RI = 0.7586, NMI = 0.5290).

**Table 3:** Prioritization performance obtained by the single controlled vocabularies and the multi-view approach

Single CV	Error of AUC	Integration of 9 complete CVs	Error of AUC
LDDB	**0.0792**	Order statistics	0.0990
eVOC	0.0852	Average score	0.0782
MPO	0.0974	Maximum score	0.0957
GO	0.1027	1-SVM *μ*_*min *_= 0	0.0620
MeSH	0.1043	1-SVM *μ*_*min *_= 0.5/N	0.0583
SNOMED	0.1129	1-SVM *μ*_*min *_= 1/N	**0.0509**
OMIM	0.1214		
Uniprot	0.1345		
KO	0.1999		

**Integration of 9 LSI**	**Error of AUC**	**Integration of 35 subset CVs**	**Error of AUC**

Order statistics	0.0645	Order statistics	0.0870
Average score	0.0382	Average score	0.0674
Maximum score	0.0437	Maximum score	0.0883
1-SVM *μ*_*min *_= 0	0.0540	1-SVM *μ*_*min *_= 0	0.1036
1-SVM *μ*_*min *_= 0.5/N	0.0454	1-SVM *μ*_*min *_= 0.5/N	0.0851
1-SVM *μ*_*min *_= 1/N	**0.0335**	1-SVM *μ*_*min *_= 1/N	**0.0625**

The experiments are repeated 20 times on random candidate gene sets and the standard deviations are all smaller than 0.01.

Comparing the results of single view data, we found that the performance varies strongly by the choice of CV. The largest error in prioritization (KO, 0.1999) was more than 2 times larger than the smallest one (LDDB, 0.0792). The discrepancy on clustering was also significant. In short, single view text model was shown fragile w.r.t. the uncertainty, which is consistent with the result in our earlier work [2].

The main finding of this approach was that combining multiple CVs in text mining improves the performance of disease gene prioritization and clustering. It is often practically difficult to predict the best candidate CV, thus the main advantage of our approach was its robustness w.r.t. to the uncertainty because the overall performance does not solely depend on any single CV and is more likely to be near-optimal.

### Combining LSI with data fusion improves prioritization performance

**Figure 2:**
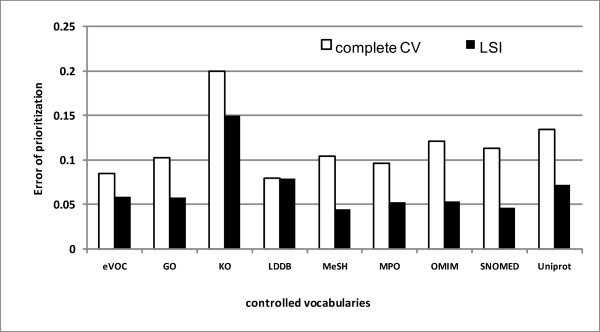
**Prioritization results obtained by complete CV profiles and LSI profiles**.

As discussed, we applied two different methods (LSI and subset CVs) to reduce the dimensionality of gene-by-term profiles. In prioritization, the effect of LSI on single view data is shown in Figure 2. LSI almost reduced the error of prioritization on all CVs (except LDDB). The best performance was obtained by MeSH-LSI (Error of AUC, 0.0453).

**Figure 3:**
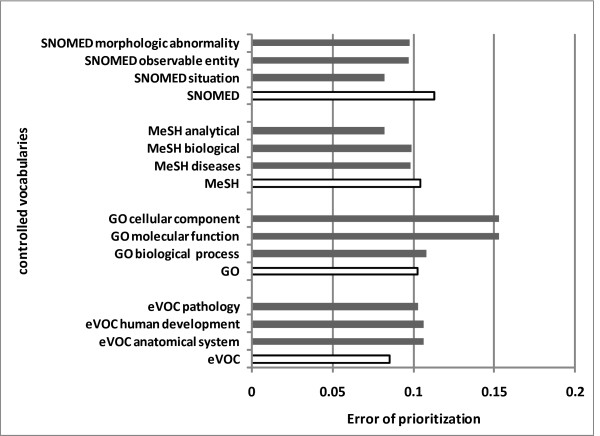
**Prioritization results obtained by complete CV profiles and subset CV profiles**.

The comparison of subset CVs with the complete CV profiles is illustrated in Figure 3. For each CV, the three best subset CV profiles are shown. On SNOMED and MeSH, the presented subset CV profiles outperformed the complete CV. However, subset CV profiles didn't work well on GO and eVOC. This is probably because SNOMED and MeSH contain vast amounts of terms, so even when split into different subsets, many redundant terms still remain. The improvement may also be ascribed to the specific descriptions about diseases and phenotypes in the subset CVs (i.e., SNOMED morphologic abnormality, MeSH disease headings, etc.). On the contrary, the number of terms in GO and eVOC is relatively small. In particular, the sub-ontologies of eVOC are orthogonal with each other so there are fewer redundant terms. After splitting, the semantic effect preserved in the ontological descriptions is lost and the performance decreases. Despite the variation of performance, the idea of splitting complete CV into subset CVs might be useful to handle textual data containing immense amount of term features. When the term number is huge, statistical feature selection techniques might not be reliable and efficient, therefore, to prune the terms with their underlying ontological structure is an efficient strategy.

**Figure 4:**
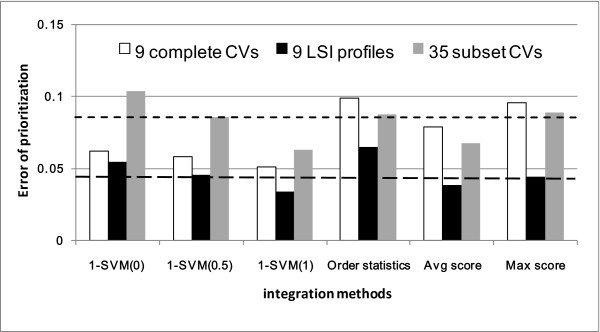
**Prioritization results obtained by multi-view data integration**. The first and the second dotted horizontal lines represent the errors of the best single complete CV profile and the best single LSI profile respectively. To prove the statistical significance between the two closest performance, we used the paired t-test to compare the Error values of the 1-SVM (1) with LSI profile integration with the values of MeSH LSI profile obtained in 20 repetitions, the p-value was 2.67e-004.

Our main interest was to integrate text models in the reduced dimensionality. We firstly combined the 9 LSI profiles, next combined all subset CV profiles. Though some CV subset profiles were observed performing better than the complete CV, as the rationale of machine learning, we should not only select the best model by validation results. Therefore, we integrated all the 30 subset CVs (6 from eVOC, 3 from GO, 7 from MeSH, and 14 from SNOMED) with the 5 other complete CVs (MPO, LDDB, OMIM, KO, and Uniprot). Result of these two approaches are compared in Figure 4. Without LSI, the 1-SVM data fusion reduced the error from 0.0792 to 0.0453. When coupling LSI with 1-SVM, the error was further reduced from 0.0453 to 0.0335. Considering the cost and effort of validating the false positive genes in lab experiments, the improvement from 0.0792 to 0.0335 is quite meaningful because it means that when prioritizing 100 candidate genes, our proposed method saves the effort of validating 4 false positive genes. Our result is also comparable to the performance of the existing systems. In the Endeavour system [24], the same disease benchmark dataset and evaluation method are implemented whereas the main differences are two aspects: Firstly, Endeavour combines one gene-by-term data (GO-IDF profile) with nine biological data sources and there is no dimensionality reduction applied. Second, Endeavour applies order statistics to integrate the rankings obtained from multiple data sources. The performance obtained in our approach is much better than Endeavour (Error = 0.0833). Moreover, our result also improves over the work of De Bie *et al*. [25]. In their approach, they use the same data sources as Endeavour and apply the 1-SVM for model integration (best performance Error = 0.0477, *μ*_*min *_= 0.5/*N*). In our approach, the data fusion method and the disease gene benchmark data are exactly the same, the improvement is therefore attributed to multi-view strategy and the LSI dimensionality reduction.

### Dimensionality reduction of gene-by-term profiles for clustering

**Figure 5:**
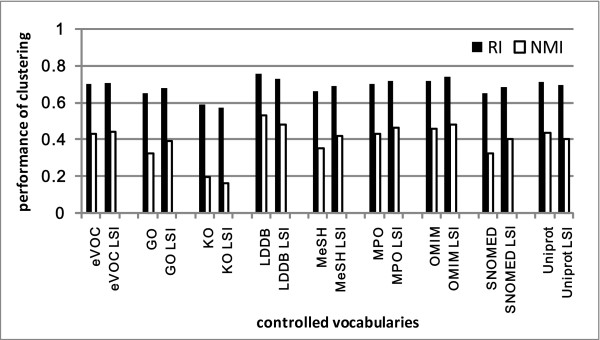
**Clustering results obtained by complete CV and LSI profiles**.

**Figure 6:**
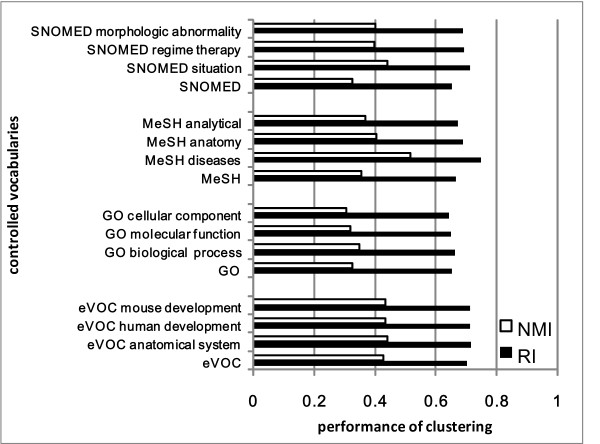
**Clustering results obtained by complete CV and subset CV profiles**.

The same LSI profiles and subset CV profiles for prioritization are also used in clustering task. As shown in Figure 5 and Figure 6, some LSI profiles were slightly better, others were slightly worse than the complete profiles. Some subset CVs performed better than the complete CV. In particular, SNOMED situation and MeSH diseases outperformed significantly the complete CVs.

**Figure 7:**
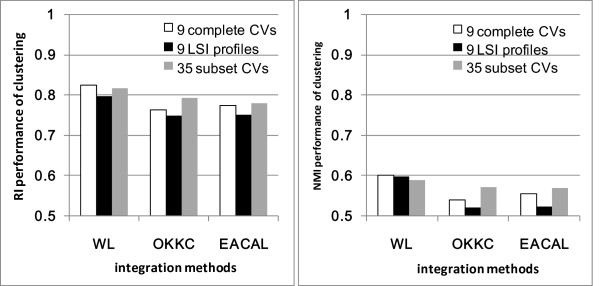
**Clustering results obtained by multi-view data integration**.

Analogue to the prioritization task, we integrated 9 complete CVs, 9 LSI profiles, and 35 subset CVs for clustering and evaluated the performance. As shown in Table 4 and Figure 7, the best result was obtained by combing 9 complete CVs with Ward linkage, OKKC (*μ*_*min *_= 0), and EACAL. Other comparing methods did not obtain better results than the best single CV.

**Table 4:** Clustering performance obtained by the single controlled vocabulary and the multi-view approach

Single CV	RI	NMI	Integration (9 CVs)	RI	NMI
LDDB	**0.7586 **± 0.0032	**0.5290 **± 0.0032	Ward linkage	**0.8236 **± 0	**0.6015 **± 0
OMIM	0.7216 ± 0.0009	0.4606 ± 0.0028	EACAL	0.7741 ± 0.0041	0.5542 ± 0.0068
Uniprot	0.7130 ± 0.0013	0.4333 ± 0.0091	OKKC(*μ*_*min *_= 0)	0.7641 ± 0.0078	0.5395 ± 0.0147
eVOC	0.7015 ± 0.0043	0.4280 ± 0.0079	MCLA	0.7596 ± 0.0021	0.5268 ± 0.0087
MPO	0.7064 ± 0.0016	0.4301 ± 0.0049	QMI	0.7458 ± 0.0039	0.5084 ± 0.0063
MeSH	0.6673 ± 0.0055	0.3547 ± 0.0097	OKKC(*μ*_*min *_= 1/N)	0.7314 ± 0.0054	0.4723 ± 0.0097
SNOMED	0.6539 ± 0.0063	0.3259 ± 0.0096	AdacVote	0.7300 ± 0.0045	0.4093 ± 0.0100
GO	0.6525 ± 0.0063	0.3254 ± 0.0092	CSPA	0.7011 ± 0.0065	0.4479 ± 0.0097
KO	0.5900 ± 0.0014	0.1928 ± 0.0042	Complete linkage	0.6874 ± 0	0.3028 ± 0
			Average linkage	0.6722 ± 0	0.2590 ± 0
			HGPA	0.6245 ± 0.0035	0.3015 ± 0.0071
			Single linkage	0.5960 ± 0	0.1078 ± 0

**Integration (9 LSI)**	**RI**	**NMI**	**Integration (35 subset CVs)**	**RI**	**NMI**

Ward linkage	0.7991 ± 0	0.5997 ± 0	Ward linkage	0.8172 ± 0	0.5890 ± 0
OKKC(*μ*_*min *_= 0)	0.7501 ± 0.0071	0.5220 ± 0.0104	OKKC(*μ*_*min *_= 0)	0.7947 ± 0.0052	0.5732 ± 0.0096
EACAL	0.7511 ± 0.0037	0.5232 ± 0.0075	EACAL	0.7815 ± 0.0064	0.5701 ± 0.0082

For integration of 9 LSI and 35 subset CVs, only the best three results are shown.

### Multi-view approach is better than merging vocabularies

**Figure 8:**
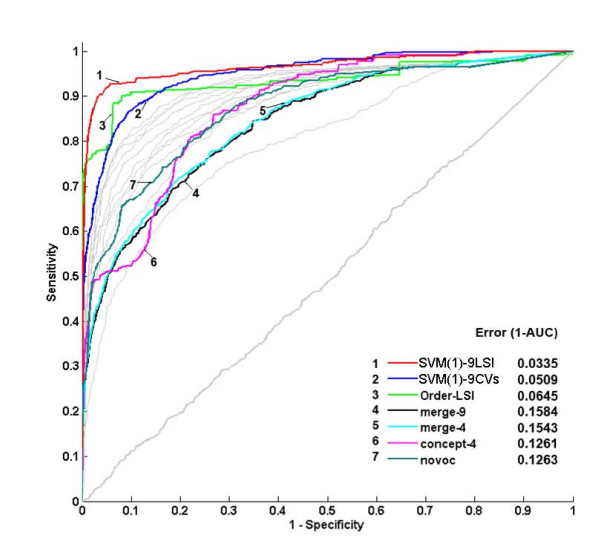
**ROC curves of prioritization obtained by various integration methods**. The light grey curves represent the single CV performance. The near-diagonal curve is obtained by the prioritization of random genes.

Our *multi-view *approach is featured by the "integration after splitting" process. As discussed in the METHOD section, an alternative approach is to merge the multiple CVs as a union CV for text mining. To evaluate this, we applied the merge-9, merge-4, novoc, and concept-4 profiles in the prioritization and clustering tasks. The ROC curves of prioritization are illustrated in Figure 8. The evaluations of clustering are listed in Table 5. Obviously, our "integration after splitting" strategy performed significantly better than the comparing methods.

**Table 5:** Clustering performance obtained by merging controlled vocabularies, concept mapping and no vocabulary indexing

Merging vocabulary	RI	NMI
merge-9	0.6321 ± 0.0038	0.2830 ± 0.0079
merge-4	0.6333 ± 0.0053	0.2867 ± 0.0085
concept-4	0.6241 ± 0.0056	0.2644 ± 0.0111
novoc	0.5630	0.0892

The merge-9, merge-4 and concept-4 profiles were clustered by K-means in 20 random repetitions and the mean values and deviations of evaluations are shown in the table. The novoc profile was only evaluated once by K-means because of the extremely high dimension and the computational burden.

### Effectiveness of multi-view demonstrated on various numbers of views

**Figure 9:**
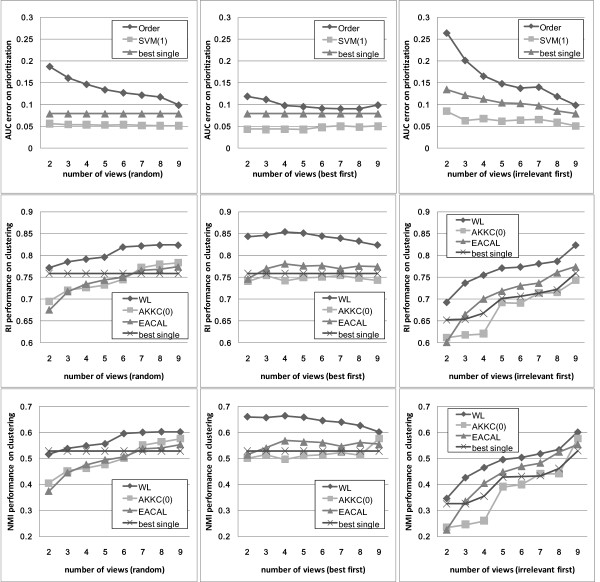
**Multi-view prioritization and clustering by various numbers of views**.

To further demonstrate the effectiveness of multi-view text mining, we evaluated the performance on various numbers of views. The number was increased from 2 to 9 and three different strategies were adopted to add the views. Firstly, we simulated a random strategy by enumerating all the combinations of views from the number of 2 to 9. The combinations of 2 out of 9 views is , 3 out of 9 is , and so on. We calculated the average performance of all combinations for each number of views. In the second and the third experiment, the views were added by two different heuristic rules. We ranked the performance of the nine views: In prioritization, the order from high to low was LDDB, eVOC, MPO, GO, MeSH, SNOMED, OMIM, Uniprot, and KO. In clustering, the order was LDDB, OMIM, Uniprot, eVOC, MPO, MeSH, SNOMED, GO, and KO. The second strategy combined best views first and increases the number from 2 to 9. In the third strategy, the irrelevant views were integrated first. The results obtained by these three strategies are presented in Figure 9. The performance of the random strategy increases steadily with the number of views involved in integration. In the best view first strategy, the performance increased and reached the ideal performance, then started to decrease when more irrelevant views are involved. The ideal performance of prioritization was obtained by combining the five best views (Error of AUC = 0.0431) by the 1-SVM method applied on averagely combined kernel. The generic integration method (order statistic) did not perform well on high dimensional gene-by-term data. The ideal performance of clustering was obtained by combining the four best views (RI = 0.8540, NMI = 0.6644) using the ward linkage method. The performance of integrating all CVs was comparable to the ideal performance, which shows that the proposed multi-view approach is quite robust to the irrelevant views. Furthermore, the merit in practical explorative analysis is that the near-optimal result can be obtained without evaluating each individual model. In the third strategy, because the combination starts from the irrelevant views first, the performance was not comparable to the random or the ideal case. Nevertheless, as shown in Figure 9, the performance of the multi-view approach was always better than the best single view involved in integration. Collectively, this experiment clearly illustrated that the *multi-view *approach is a promising and reliable strategy for disease gene identification.

### Effectiveness of multi-view demonstrated on disease examples

To understand why the improvements took place when combining the multiple views, we presented two case studies. The first example was taken from the prioritization of MTM1, a gene relevant to myopathy disease. In the disease benchmark data set, myopathy contains 41 relevant genes so we built the disease model using the other 40 genes and left MTM1 out with 99 random candidate genes for validation. To compare the rankings, only in this experiment, the 99 random candidate genes were kept identical in different views. In Table 6, we list the ranking positions of MTM1 and the "false positive genes" ranked before it. On LDDB, three "false positive genes" (C3orf1, HDAC4, and CNTFR) were ranked higher than MTM1. To investigate the terms causing this, we sorted the terms by their correlation scores. The correlation scores were computed between the gene-by-term profile of the candidate gene and the prioritization model (the average gene-by-term profile of the genes in the training set). The real relevant gene MTM1 was highly correlated on terms like "muscle, muscle weak, skeletal, hypotonia, growth, lipid", and so on. C3orf1 was ranked at the top because of the high correlation on terms such as "skeletal, muscle, heart", and so on. HDAC4 was ranked second on terms like "muscle, heart, calcium, growth", and some others. CTNFR was ranked as the third due to terms such as "muscle, heart, muscle weak, growth", and so on. Nevertheless, according to our knowledge none of these three genes (C3orf1, HDAC4, and CNTFR) is actually known to cause any disease. Escarceller *et al*. [43] show that C3orf1 seems to be enhanced in heart and skeletal muscle, but there is no evidence about its relevance to the disease. HDAC4 is found in the work of Little *et al*. "as a specific downstream sbstrate of CaMKIIdeltaB in cardiac cells and have broad applications for the signaling pathways leading to cardiac hypertrophy and heart failure" [44]. In the papers of Glenisson *et al*. [45] and Cohen *et al*. [46], HDAC4 is found to have a role in muscle, which means it might be a good candidate but it has not been directly proved as a relevant gene. For CNTFR, it has been found that in heterozygotic mice inactivation of the CNTFR leads to a slight muscle weakness [47]. In the papers of Roth *et al*. [48] and De Mars *et al*. [49], CNTFR is shown related to muscular strength in human. Collectively, although there is no evidence that these 3 genes found by LDDB are disease causing factors, the prioritization result was still meaningful because they have similar correlated terms as the real disease gene MTM1. Especially, HDAC4 and CNFR seem to be nice candidates to muscular disorder. Though LDDB ranked 3 "false positive" genes higher than the real disease relevant gene, eVOC and GO ranked the real gene MTM1 as the top candidate. On eVOC, the most important correlated terms were "muscle, sever, disorder", and so on. On GO, the highly correlated terms were "muscle, mutation, family, sever", and others. In multi-view result obtained using the 1-SVM (*μ*_*min *_= 1/*N*), the ranking of LDDB was complemented by eVOC and GO thus MTM1 was ranked as the top gene.

**Table 6:** Prioritization of the myopathy disease relevant gene MTM1 by different CVs and multi-view approach

CV	Ranking position	false positive genes	correlated terms
LDDB	1	C3orf1	muscle, heart, skeletal
	2	HDAC4	muscle, heart, calcium, growth
	3	CNTFR	muscle, heart, muscle weak, growth
	**4**	**MTM1**	muscle, muscle weak, skeletal, hypotonia, growth, lipid

eVOC	**1**	**MTM1**	muscle, sever, disorder, affect, human, recess

MPO	1	HDAC4	muscle, interact, protein, domain, complex
	2	HYAL1	sequence, human, protein, gener
	3	WTAP	protein, human, sequence, specif
	4	FUT3	sequence, alpha, human
	...		
	**15**	**MTM1**	myopathy, muscle, link, sequence, disease, sever

GO	1	**MTM1**	muscle, mutate, family, gene, link, seqeuence, sever

MeSH	1	HYAL1	human, protein, clone, sequence
	2	LUC7L2	protein, large, human, function
	**3**	**MTM1**	myopathy, muscle, mutate, family, gene, missens

SNOMED	1	S100A8	protein, large, human, function
	2	LUC7L2	protein, large, human, function
	3	LGALS3	human, protein, express, bind
	...		
	**23**	**MTM1**	muscle, mutate, family, gene, link

OMIM	1	HDAC4	muscle, interact, protein, bind
	2	MAFK	sequence, protein, gene, asthma relat trait
	3	LUC7L2	protein, large, function, sequence
	4	SRP9L1	sequence, protein, length, function
	...		
	**50**	**MTM1**	muscle, family, gene, link, sequence, disease, sever, weak

Uniprot	1	**MTM1**	gene, protein, function

KO	1	S100A8	protein, bind, complex, specif, associ, relat
	2	PRF1	specif, protein, contain, activ
	...		
	**56**	**MTM1**	protein, large, specif, contain

Multi-view	**1**	**MTM1**	
	2	HDAC4	
	3	CNTFR	

The second example was taken from the clustering task of genes relevant to breast cancer and muscular dystrophy, where each disease contains 24 non-overlapping genes, as shown in Table 7. We list the confusion tables and mis-partitioned genes of each single view in Table 8. As illustrated, single views all produced some mis-partitioned genes. In the multi-view approach (ward linkage), all the genes were correctly partitioned to the correct disease labels.

**Table 7:** Genes relevant to breast cancer and muscular dystrophy

Disease	Breast Cancer	Muscular Dystrophy
relevant genes	AR	CAPN3
	ATM	CAV3
	BCAR1	COL6A1
	BRCA1	COL6A3
	BRCA2	DMD
	BRIP1	DYSF
	BRMS1	EMD
	CDH1	FKRP
	CHEK2	FKTN
	CTTN	FRG1
	DBC1	LAMA2
	ESR1	LMNA
	NCOA3	MYF6
	PHB	MYOT
	PPM1D	PABPN1
	RAD51	PLEC1
	RAD54L	SEPN1
	RB1CC1	SGCA
	RP11-49G10.8	SGCB
	SLC22A18	SGCD
	SNCG	SGCG
	TFF1	TCAP
	TP53	TRIM32
	TSG101	TTN

**Table 8:** Clustering breast cancer and muscular dystrophy relevant genes by different CVs and the multi-view approach

CV	Breast Cancer	Muscular Dystrophy	mis-partitioned genes
LDDB	22	2	RP11-49G10.8, FKTN
	0	24	

eVOC	22	2	RP11-49G10.8, FKTN
	7	17	LMNA, COL6A1, MYF6, CHEK2, SGCD, FKRP, DMD

MPO	23	1	RP11-49G10.8
	1	23	SGCD

GO	23	1	RP11-49G10.8
	7	17	LMNA, COL6A1, MYF6, CHEK2, SGCD, FKRP, DMD

MeSH	23	1	RP11-49G10.8
	2	22	SGCD, COL6A3

SNOMED	24	0	
	6	18	LMNA, COL6A1, MYF6, TRIM32, SGCD, DMD

OMIM	24	0	
	1	23	SGCD

Uniprot	24	0	
	4	20	MYF6, CHEK2, SGCD, FKRP

KO	19	5	SLC22A18, RP11-49G10.8, FKTN, PABPN1, CAPN3
	6	18	PPM1D, MYF6, SGCD, FKRP, COL6A3, DYSF

Multi-view (WL)	24	0	
	0	24	

## Discussion

The merit of our approach lies in the conjunction of data fusion methods with dimensionality reduction techniques and its application on biomedical text mining to solve the gene prioritization and clustering problems.

The issue of model integration has already been investigated in several text mining applications. The notion of *multi-view *has been proposed by Bickel and Scheffer [50] in web document clustering analysis to combine intrinsic view (text based similarity) and extrinsic view (citation link based similarity) of web pages. We refer the term *multi-view *to denote the gene-by-term profiles represented by different CVs. Neveol *et al*. [6] combine three different methods (dictionary lookup, post-processing rules and NLP rules) to identify MeSH main heading/subheading pairs from medical text. Chun *et al*. [51] develop an integrative system to extract disease-gene relations from MEDLINE. Jimeno *et al*. [7] combine three methods (dictionary look-up, statistical scoring, and MetaMap) to recognize disease names on a corpus of annotated sentences. Gaulton *et al*. [5] adopt 3 different ontologies and 8 data sources in the CAESAR system to annotate human genes as disease associated candidates. When annotating multiple data sources with different relevant terms from ontologies, each gene can get multiple scores of relevance with the input text query. CAESAR combines the scores using 4 basic methods: maximum, sum, normalized average, and a transformed score penalized by the number of genes annotated in a given data source. Our approach differs from CAESAR by exploiting all the relevant MEDLINE abstracts so the gene-by-term profiles are retrieved from vast amounts of free-text information in literature. We have shown that these profiles can solve different text-based computational problems such as prioritization and clustering. Yamakawa *et al*. [52] combine 3 different sources (GO, Locuslink, and HomoloGene) to create gene list annotated with GO terms. Then, a decomposition method (ETMIC situation decomposition) is applied to extract multiple aspects information from the target gene list, resulting in several bipartite graphs describing the relationships between small subset of genes and the GO terms. Their approach has the same motivation as ours, that is, to obtain more refined characteristics of genes separated in different aspects (views). They however haven't shown how multi-aspect gene annotations can improve the effectiveness of biomedical knowledge discovery.

In the past decade, many computational approaches have been developed to prioritize disease candidate genes using textual data [53-57]. To obtain thorough evidences for complex genetic diseases, many efforts focus on combining computational models learned from multiple data sources [24-26,58,59]. In particular, De Bie *et al*. [25] formulate gene prioritization as a novelty detection task and combine textual data with biological data in a kernel optimization framework. We used the same 1-SVM method to integrate multi-view text data and further intervene data fusion with dimensionality reduction. We have shown that it leads to better performance in prioritization.

Clustering by multiple (heterogeneous) data sources is an ongoing topic with many interests. Recently, Wolf *et al*. [60] have investigated the memory persistence (long term or short term memory) of bacteria by observing a strain of Bacillus subtilis at different experimental conditions and developmental times. These multiple observations are then analyzed by clustering to quantify the mutual information the bacterium "remembers" at different stages. Some other approaches address consensus clustering to combine multiple partitions generated on a single dataset, for instance, the analysis of microarray data by Monti *et al*. [61] and Yu *et al*. [62]. Asur *et al*. [63] adopt consensus clustering methods to combine matrices generated by 3 different types of measurements (topological measure, mutual information measure and GO annotations) to cluster Protein-Protein Interaction networks. Lange and Buhmann [35] merge similarity matrices and phrase multi-source clustering as a non-negative matrix factorization problem. The kernel fusion problem for clustering is connected to many active works in machine learning and optimization, for instance, the framework of linear kernel fusion for binary supervised learning task proposed by Lanckriet *et al*. [64] and Bach *et al*. [65] and its extension to multi-classes problem proposed by Ye *et al*. [41]. Sonnenburg *et al*. simplify the computational burden of kernel fusion by Semi-infinite programming (SIP) [40]. On the basis of kernel fusion, Chen *et al*. [66] propose a clustering algorithm called nonlinear adaptive distance metric learning as an analogue of Lanckriet et al.'s statistical framework for clustering. Yu *et al*. (Yu *et al*.: Optimized data fusion for kernel K-means clustering, submitted) propose a clustering algorithm, OKKC, for heterogeneous data fusion and combine text mining data and bibliometrics data to explore the structure mapping of journal sets [37]. In this paper, we systematically evaluate and compare 12 representative algorithms from two main approaches, ensemble clustering and kernel fusion, to combine the multi-view data. Our experimental result shows that ward linkage, OKKC, and EACAL perform better than other methods. The number of disease genes in our benchmark data is imbalanced, which may partially affect the evaluation of clustering results (see Additional file 1).

The interpretation of text based prioritization is limited by LSI, whose latent factors cannot be easily attributed to the terms affecting the prioritization. When combining multi-view data by K-means and ensemble algorithms (individual partition created by K-means), to estimate the optimal cluster numbers is also difficult because the number of clusters is predefined. The statistical evaluations of clustering quality which is used to indicate the optimal cluster number on single data set are not always reliable for data fusion because they may differ in heterogeneous data sources. To circumvent this problem, one may relax the K-means clustering as a spectral clustering [67] thus the optimal cluster number can be investigated from the eigenspectrum. To estimate the optimal cluster number in hierarchical clustering is easier, because it can be estimated by checking the dendrogram. Another limitation in our clustering approach is the ignorance of overlapping genes despite of the fact that a gene may be biologically relevant to several topics (i.e., diseases, functions, processes, etc.). Therefore, how to apply "soft clustering" techniques to obtain partitions containing overlapping genes will be the main topic of our future work. The notion of *multi-view *text mining has the potential of incorporating models varied by other parameter. For example, instead of using curated GeneRIF as the mapping of genes to publications, one can detect gene names expressed in the text automatically by natural language processing (NLP) and create new gene profiles according to this mapping. One can also retrieve the relationships of genes from literature, or refer to interaction networks and produce new view specified about relationships of genes. Combining these views will undoubtedly lead to significant and thorough insight about the associations between diseases and genes.

## Conclusions

We have presented the approach of combining *multi-view *text mining models to obtain precise identification of disease relevant genes. These views were specified by multiple controlled vocabularies derived from different bio-ontologies. Using these vocabularies, we have indexed the MEDLINE titles and abstracts relevant to GeneRIF and have obtained a series of gene-by-term profiles. To demonstrate the effectiveness of our approach, we have combined these profiles and evaluated them on two fundamental problems: gene prioritization and clustering. Experimental results have shown that the performance obtained on the *multi-view *approach is significantly better than the single-view data. Nonetheless, the selection of the appropriate integration algorithm was nontrivial. We have cross-compared 4 algorithms in prioritization and 12 algorithms in clustering on a disease benchmark data set containing 29 diseases. In prioritization, the combination of the 1-SVM with LSI performed the best; in clustering, the ward linkage applied on the uniform combination of kernels performed better than other methods.

Second, we have integrated dimensionality reduction of individual data source in the data fusion framework. To tackle the very high dimensionality of text mining data, we have applied LSI, a popular reduction technique in information retrieval, on gene-by-term profiles. Alternatively, we have also pruned the vocabularies according to the hierarchical structures of the bio-ontologies where they were derived. In this way, the gene-by-term profiles specified by a complete CV have been further separated as several subset CV profiles. In some experiments, the LSI and the subset CV profiles have obtained better performance than the complete CV.

Third, we have substantiated the rationale of the proposed "integration after splitting" by comparing three other methods such as vocabulary integration, concept mapping, and no vocabulary indexing. Experiments and validation results have clearly indicated that the proposed *multi-view *approach is a promising strategy.

## Authors' contributions

All authors conceived the project and design. SY performed text mining, programmed the algorithms, analyzed the data and wrote the paper. LCT collected the disease benchmark dataset, programmed the order statistics method for prioritization model integration, and investigated the false positive genes in prioritization. BDM and YM are the promoters of SY and LCT. All authors read and approved the manuscript.

## Supplementary Material

Additional file 1 **Discussion about the effect of class imbalance in clustering evaluation**. extended discussion about the data and the result. Click here for file
