# Supplementary material for: Gene prioritization and clustering by multi-view text mining
Source: BMC Bioinformatics. 2010 Jan 14;11:28. doi: 10.1186/1471-2105-11-28 (PMC3098068; doi:10.1186/1471-2105-11-28)
Supplement: Additional file 1 — Discussion about the effect of class imbalance in clustering evaluation. extended discussion about the data and the result. [file 1471-2105-11-28-S1.PDF]

Gene prioritization and clustering by multi-view text mining

Shi Yu, Leon-Charles Tranchevent, Bart De Moor, Yves Moreau

Additional file 1: Discussion about the effect of class imbalance in clustering evaluation

We first list the numbers of disease relevant genes in our benchmark data set in Figure 1.

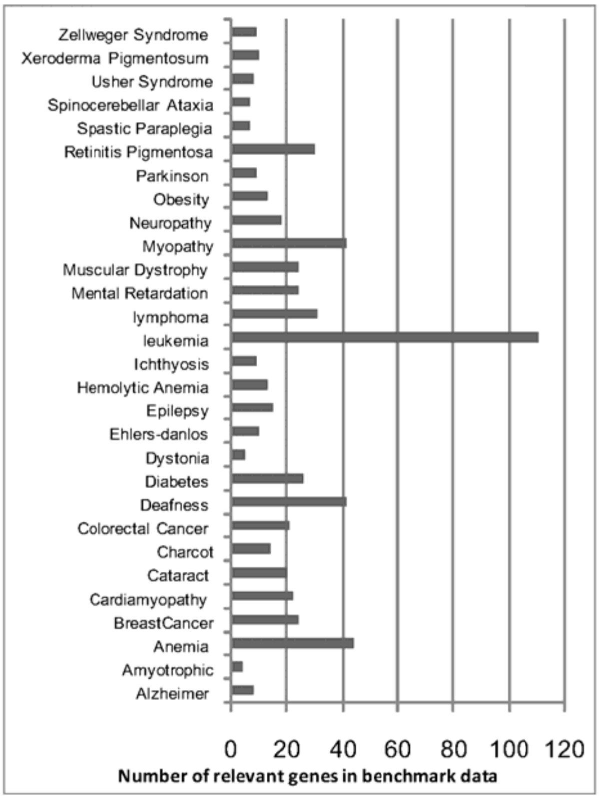

Figure 1 The numbers of disease relevant genes in benchmark data

As shown, the numbers of different disease relevant genes are quite imbalanced. Therefore, the clustering experiments designed in our approach actually evaluated results by the imbalanced disease labels. To indicate the degree of imbalance as a numerical value, we computed the ratio between the numbers of disease relevant genes for each clustering task. We set the small number as the numerator so this ratio is always no larger than 1. The average ratio over 406 paired tasks is 0.5026, which means in our clustering tasks, the genes relevant to one disease are averagely twice as much as the one relevant to another disease.

This imbalance problem of disease labels affected our clustering evaluation. Next, we investigated the same ratio in clustering results. Since the cluster number was predefined (K=2), we computed this ratio for all the clustering algorithms applied in our experiment. The ratios are

all obtained by multi-view approach (combing 9 complete CVs) and the results are listed in the following table:

Table 1 The ratios of class imbalance observed on all clustering algorithms in the mult-view approach

Algorithm	Ratio
**Ward linkage**	0.4953
**Single linkage**	0.0588
**Average linkage**	0.1543
**Complete linkage**	0.2384
**AKKC**	0.6452
**K-means**	0.6795
**EACAL**	0.6481
**QMI**	0.7166
**adacVote**	0.4609
**MCLA**	0.6971
**CSPA**	0.9225
**HGPA**	0.891

As shown, the automatic partitions obtained by the hierarchical clustering methods (SL, WL, CL and AL) are also imbalanced (all smaller than 0.5). On the contrary, other algorithms are more likely to provide balanced partitions thus their ratios are much closer to 1. As the matter of fact, the ratio of WL is the most similar one to the ratio of the disease labels. As a “caveat”, the skewed distribution of disease genes partially affected the evaluations, which also explained why WL performed much better than K-means and other algorithms in our approach.
